# Accidental Explantation of a Cochlear Implant in a Child Who Developed Cholesteatoma as a Late Complication of Cochlear Implantation

**DOI:** 10.1155/2020/6353706

**Published:** 2020-10-09

**Authors:** Wong Kein Low, Wan Ni Pok, Win Nie Ng, Judy Tan

**Affiliations:** ^1^Novena ENT Head and Neck Surgery Specialist Centre, Singapore; ^2^Duke-NUS Graduate Medical School, Singapore; ^3^Department of Radiology & Nuclear Medicine, Mount Elizabeth Novena Hospital, Singapore

## Abstract

**Introduction:**

Although rare, cholesteatoma can develop as a late complication of cochlear implantation. The electrode array may then be exposed in the external auditory canal surrounded by cholesteatoma debris. *Case Report*. The cochlear implant of a child was inadvertently explanted by a clinician during a routine aural toilet procedure. The child had previously reported recurrent ear infections, pain, and unexplained implant function degradation. Reimplantation was carried out 2 days later with good postoperative hearing results. Part of the electrode array was observed to be embedded in cholesteatoma. Postreimplantation recovery was complicated by a breakdown of the blind-sac. *Discussion*. Clinical indicators that could alert the clinician to the possibility of this late complication include recurrent infections, presence of keratotic debris in the external auditory canal, unexplained implant function degradation, and nonauditory stimulation. Although this patient managed to achieve excellent postreimplantation hearing outcomes, a delay in reimplantation surgery following explantation could possibly compromise successful reinsertion of the electrode array. External ear canal overclosure without mastoid cavity obliteration has merit in facilitating CT scan surveillance, but it may increase the risk of the blind-sac breaking down. This case also illustrated how the electrode array could have facilitated propagation of the cholesteatoma from the middle ear to the mastoid.

**Conclusion:**

If aural toilet is required in the implanted ear of a cochlear implant recipient, any complaint of hearing change, pain, or discharge should alert the clinician of the possibility of cholesteatoma developing. It warrants prompt evaluation by an experienced otologist in order to prevent accidental explantation. *Keywords*. Cochlear implant, cochlear implant complications, chronic suppurative otitis media, cholesteatoma, reimplantation, blind-sac, external auditory canal overclosure, mastoid cavity obliteration.

## 1. Introduction

The prevalence of cholesteatoma developing in patients after cochlear implantation ranges from 4.8 to 12.2% [1]. These may include recurrent/persistent disease in postimplanted patients who had prior cholesteatoma [2]. It is, indeed, rare to have cholesteatoma developing as a complication of cochlear implant surgery itself [3, 4].

Reimplantation, if carried out immediately after explantation, frequently results in good postoperative hearing outcomes [5, 6]. Delays in reimplantation, however, could possibly be complicated by soft tissue formation leading to obstruction of the cochleostomy opening [7].

This report describes and discusses a child who developed cholesteatoma as a late complication of cochlear implantation. Following accidental explantation of the cochlear implant, reimplantation was subsequently carried out as a delayed procedure.

## 2. Case Report

KIE was a nine-year-old female foreigner first seen by our centre at the age of 2 years for delayed speech and language development. There was an antenatal history of maternal rubella infection in the first trimester. Clinical examination of the external ear canals and tympanic membranes, then, was unremarkable. She was diagnosed with bilateral sensorineural hearing loss at a profound level for which cochlear implantation was recommended. She had right cochlear implant surgery (Advanced Bionics HiRes 90K HiFocus 1J) performed at another centre when she was 2.5 years of age. After surgery, she returned to our centre for regular audiological and auditory-verbal therapy follow-ups. As she continued medical follow-up with a local otolaryngologist from her hometown overseas, we did not possess records of her medical condition including the status of her eardrums.

Audiologically, she was doing fine until at 3 years after implantation when she started to experience nonauditory stimulation during mapping. Whenever electrode 16 (the most proximal electrode) was stimulated, she would experience throat irritation and coughing episodes. At a subsequent review 6 months later, the nonauditory symptoms resolved, but she lost her auditory perception at electrode 16 (even at elevated M-levels of more than 400CU). Six months later, she reported pain and discomfort upon stimulation of electrodes 15 and 16. At 5 years after implantation, her score on a speech test was 80% without lip reading.

At 6 years after implantation, she returned for an urgent medical consult. She gave the history of suddenly experiencing severe pain and complete loss of hearing during an aural toilet procedure in the clinic the day before. Prior to that, she had been experiencing recurrent itch and discharge from the right ear for a few months but was still able to hear with the implant.

**Figure 1:**
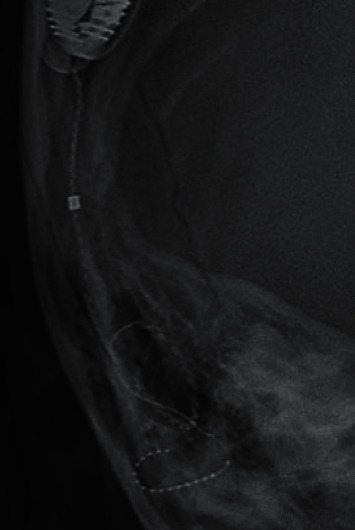
X-ray of the right mastoid (frontal view) showing the implant electrode array possibly coiled up in the region of the external auditory canal.

**Figure 2:**
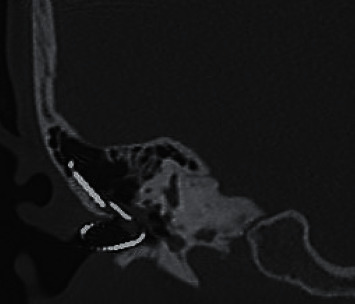
Coronal CT scan of the right temporal bone confirming explantation had taken place with the electrode array coiled up in the external auditory canal.

On examination, the tip of the electrode array could be seen in the external ear canal (EAC) (Supplementary Fig.). The eardrum appeared to be perforated with the presence of cholesteatoma debris in the middle ear and EAC. Taken together, the clinical impression, then, was that inadvertent explantation had taken place during the aural toilet procedure. The electrode array was probably exposed in the EAC secondary to the development of cholesteatoma as a complication of cochlear implantation. Indeed, X-ray and CT scan performed on the same day confirmed that explantation had taken place with the electrode array lying in the EAC (Figures 1 and 2).

The family was counseled accordingly. It was recommended that the cholesteatoma be eradicated and that a new device be reimplanted. Although these could be performed in 1 stage, the possibility of a 2-stage procedure was discussed. Radical mastoidectomy with overclosure of the EAC, as well as the pros and cons of mastoid cavity obliteration with abdominal fat, was also discussed. The possibility of failure to reinsert the replacement electrode array and the option of a second side cochlear implant on the opposite (left) ear were highlighted. The parents expressed preference for the avoidance of an additional abdominal wound for fat grafting and opted for a second side implant.

**Figure 3:**
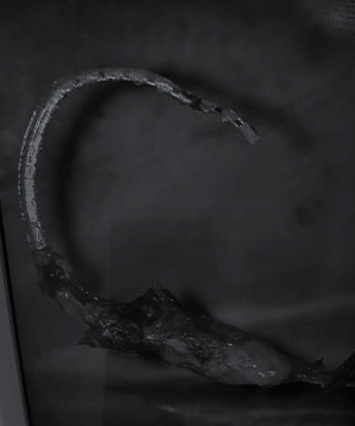
Cholesteatoma diseased tissue embedding a part of the electrode array.

**Figure 4:**
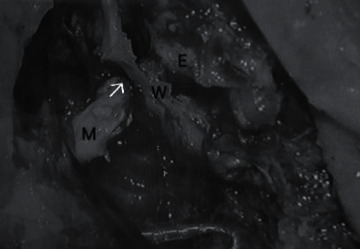
The right external ear canal and mastoid cavity separated by the bony posterior canal wall W is shown with the explanted electrode array in situ. Cholesteatoma diseased tissue which is embedding a part of the explanted electrode array is seen in the middle/external ear E and mastoid M and is connected through the posterior tympanotomy (arrow).

Reimplantation surgery was carried out 2 days later. Intraoperatively, cholesteatoma was seen in the middle ear. Embedding the electrode array, diseased tissue extended into the mastoid cavity through posterior tympanotomy (Figures 3 and 4). Histology of a sample of this tissue confirmed cholesteatoma with lamellar keratinaceous debris, strips of stratified squamous and fibrotic stroma with inflammation, and dystrophic calcification. With a canal wall down mastoidectomy, complete clearance of gross disease could be achieved. Soft tissue had formed around the cochleostomy site resulting in obstruction of the cochleostomy opening. Fortunately, following its removal with a pair of microscissors, the cochleostomy opening could be restored which permitted successful reinsertion with the same implant model.

A blind sac was created by overclosure of EAC. The EAC skin was noted to be thinned and friable, and the nearby soft tissue observed to be scarred from the previous cochlear implant surgery. As planned, the surgery was completed without further mastoid cavity obliteration.

The second side cochlear implantation (Advanced Bionics HiRes 90K HiFocus Midscala) was carried out in the left ear without any problems.

The blind sac was initially intact but started to breakdown about 3 weeks postsurgery. As it did not heal with medical treatment, she underwent reparative surgery with abdominal fat grafting 1 month later. The blind sac was healthy since and had remained so at least 2 years after surgery.

The right implant was activated 2 weeks after reimplantation and the left implant 3 months after that. All the electrodes could be activated with mapping parameters on both sides falling within normal limits. The adaptation to progressive maps of increasing loudness was well tolerated with no further nonauditory stimulation. At 1 year after surgery, she achieved a 100% speech test score on her reimplanted side and 63% on the opposite side. Aided hearing thresholds of 20 dB from 250 Hz to 8000 Hz were attained on both sides. With her new implants, she experienced increased awareness of very soft sounds and was doing well in mainstream school. She had also been swimming regularly without any problems.

## 3. Discussion

Electrode array extrusion into the EAC from the tympanic membrane and/or posterior wall of the bony canal is an uncommon complication [8, 9]. This could be a sequelae of damage to these structures during the initial cochlear implant surgery resulting in iatrogenic defects or due to excessive thinning of the posterior bony canal wall leading to subsequent breakdown [4].

As illustrated by this case, the development of cholesteatoma following cochlear implant surgery is another way where the electrode array could be exposed in the EAC. The prevalence of cholesteatoma developing in postimplanted patients ranges from 4.8 to 12.2% [1]. These may include recurrent/persistent disease in postimplanted patients who had prior cholesteatoma [2]. It is rare to have cholesteatoma developing as a complication of cochlear implantation itself [3, 4].

The etiopathogenesis of cholesteatoma is controversial. Olszewska et al. [10] stated that Habermann in 1888 and Bezold in 1890 proposed the epithelial immigration or invasion theory where squamous epithelium migrate from the margins of tympanic membrane perforations into the middle ear spaces with infection possibly playing a role [10]. The development of cholesteatoma in postimplant patients could be the result of a surgical breach of the tympanic membrane and/or EAC during the implantation surgery [4]. The present case revealed cholesteatoma diseased tissue wrapped around the electrode array and appeared to have propagated along the array from the middle ear through the posterior tympanotomy into the mastoid cavity (Figures 3 and 4). Biofilms containing bacterial colonies had been demonstrated to adhere to implanted cochlear devices [11]. It will be interesting to know if these could have contributed to the disease process.

KIE started to experience degradation of implant function 3 years after implantion. Activation of electrode 16 (the most proximal electrode) triggered throat irritation and cough. These suggested that partial electrode array extrusion from the cochlear could have taken place, with the exposed extracochlear electrode stimulating the Cranial Nerve X innervation of the EAC. It is also noteworthy that exposure of the electrode array in the EAC is not necessarily obvious, being embedded in cholesteatoma debris. Hence, recurrent infections, keratotic debris in the EAC, unexplained implant function degradation, and nonauditory stimulation should alert the clinician of the possibility of this complication so that accidental explantation could be averted.

For the reimplantation surgery, a canal wall down procedure was performed followed by the creation of a blind sac by overclosure of the EAC. In a meta-analysis study, it was found that overclosure of the EAC at the same time as cochlear implantation led to significantly fewer complications when compared to maintaining a canal wall down mastoid cavity with soft tissue coverage of the electrode array [12]. Vincenti et al. [13] highlighted a patient who had postoperative breakdown of the blind sac and recommended rigorous surgical techniques to achieve EAC overclosure and mastoid obliteration in cochlear implantation after radical mastoidectomy [13].

EAC overclosure could also be complicated later by cholesteatoma developing from squamous epithelium inadvertently left behind during the surgery [14]. As surveillance using MRI scan can be difficult in cochlear implant recipients, overclosure of the EAC without mastoid obliteration had been advocated based on the belief that an air-filled mastoid cavity would be maintained to facilitate surveillance using the CT scan [15]. Furthermore, KIE had prior cholesteatoma and, therefore, was at risk (albeit very low) for the development of recurrent/persistent disease. Her parents also preferred not to have an obliterated mastoid as it would necessitate an additional surgical wound to harvest the fat graft. Hence, as advocated by Xenellis et al. [16], a canal wall down mastoidectomy and EAC overclosure without cavity obliteration were planned at the outset [16]. Unfortunately, it was observed intraoperatively that the EAC skin was thinned and friable, probably as a result of previous recurrent infections. It was also noted that the soft tissues in the vicinity were violated and scarred from the previous cochlear implant surgery. Together, these could have compromised the creation of a blind sac which was sufficiently robust without the need for additional support of an underlying graft tissue. Indeed, the blind sac broke down subsequently necessitating further reparative surgery using an abdominal fat graft to obliterate the mastoid cavity. It can be argued that this second surgery could have been avoided if the cavity obliteration procedure had been carried out in the first place during the initial surgery.

KIE managed to achieve good hearing outcomes after reimplantation, which was consistent with the reimplantation experiences of some other authors [5, 6]. Although reimplantation was technically possible, the first procedure always provided the optimal surgical environment [17]. Explantation followed by delayed reimplantation might be complicated by compromised cochlear patency [7]. Roland et al. [18], therefore, cautioned that the attainment of good postreimplantation hearing outcomes could not be guaranteed [18]. In staged reimplantation, reinsertion could be facilitated if the electrode was retained in situ as a lumen-keeper until reimplantation could take place [19]. As this was clearly not possible in this case, a complex case of cochlear luminal obliteration could have jeopardised a successful reimplantation surgery. It was fortunate that, after removing soft tissue by sharp dissection around the cochleostomy site, a patent cochlear lumen could be identified which enabled reimplantation to proceed smoothly.

## 4. Conclusions

Cholesteatoma development can occur as a late complication of cochlear implant surgery with the electrode array being exposed in the EAC. Embedded in cholesteatoma debris, its exposure is not necessarily obvious which could result in accidental explantation during aural toilet of the EAC. In a cochlear implant recipient, any complaint of hearing change, pain, or discharge should alert the clinician of the possibility of this complication. EAC overclosure without mastoid cavity obliteration has merit in facilitating CT scan surveillance but may increase the risk of the blind sac breaking down. Although reimplantation following accidental explantation can possibly be successful with excellent postreimplantation hearing results, there is a risk that cochlear patency be significantly compromised making reimplantation difficult or even impossible.

## Data Availability

No data were used in this study.

## Supplementary Materials

Supplementary Materials Supplementary figure: External auditory canal of the right ear showing the tip of the electrode array with cholesteatoma debris in the background.
