# Supplementary material for: Accidental Explantation of a Cochlear Implant in a Child Who Developed Cholesteatoma as a Late Complication of Cochlear Implantation
Source: Case Rep Otolaryngol. 2020 Oct 9;2020:6353706. doi: 10.1155/2020/6353706 (PMC7568801; doi:10.1155/2020/6353706)
Supplement: Supplementary Materials — Supplementary figure: External auditory canal of the right ear showing the tip of the electrode array with cholesteatoma debris in the background. [file 6353706.f1.docx]

**
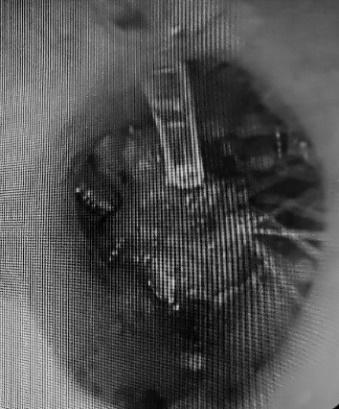
**

Supplementary Fig . External auditory canal of the right ear showing the tip of the electrode array with cholesteatoma debris in the background
